# DNA Methylation and TMPRSS2-ERG Fusion Status in Patients with Prostate Cancer from The Republic of North Macedonia

**DOI:** 10.2478/bjmg-2026-0014

**Published:** 2026-10-14

**Authors:** A Trifunovski, A Gavrilovska-Brzanov, S Dohchev, J Janculev, D Trajkovski, I Vujicic, G Severova Andreevska, M Ambardjieva, E Dodikj

**Affiliations:** University Clinic of Urology, Medical Faculty, University of “Ss. Cyril and Methodius”– Skopje, R. N. Macedonia; University Clinic for Traumatology, Orthopedic Diseases, Anesthesia, Reanimation, Intensive Care and Emergency Centre, Medical Faculty, University of “Ss. Cyril and Methodius”– Skopje, R. N. Macedonia; University Clinic of Nephrology, Medical Faculty, University of “Ss. Cyril and Methodius”– Skopje, R. N. Macedonia; University Clinic for Surgical Diseases “St. Naum Ohridski, Medical Faculty, University of “Ss. Cyril and Methodius”– Skopje, R. N. Macedonia; Department of Internal Medicine, Cardiology Unit, General Hospital-Kumanovo, North Macedonia

**Keywords:** DNA methylation, TMPRSS2-ERG, prostate cancer

## Abstract

Prostate cancer is the second leading cause of male deaths in the Western world and the most commonly detected cancer in males. Recent studies have pointed out that DNA methylation and TMPRSS2-ERG fusion transcript, have a high presence among prostate tumor cells. It is estimated that these two molecular mechanisms could be used as potential markers for early detection of malignancy, and/or progression of prostate cancer disease.

The study aimed to test the presence of the DNA methylation and TMPRSS2-ERG fusion transcript in patients with prostate cancer, as a baseline database for further genetic examination. This prospective clinical study focused on a random sample of 88 patients with prostate carcinoma. For the detection of methylation status in 26 tumor suppressor genes (*TP73, CASP8, VHL, RARB, MLH1, RASSF1, FHIT, APC, SLC9A2-2, ESR1, CDKN2A, CDKN2B, DAPK1, KLLN, PTEN, CD44, GSTP1, ATM, CADM1, CDKN1B, CHFR, BRCA2, CDH13, HIC1, BRCA1 and TIMP3*), in CaP patients the Multiplex Ligation-Dependent Probe Amplification (MLPA) analyses was performed. All samples were conducted on a Real-time PCR analysis for the detection of the TMPRSS2-ERG fusion transcript. Additionally, the data from the histopathology results of tissues, and level of prostate-specific antigen (PSA) in blood were used.

Genome-wide DNA methylation profiles were detected in 10 out of 26 genes: *GSTP1*was found in 53,4 % of samples, *RARB* in 44,32%, *RASSF1* in 63,6%, *APC* in 42%, *CDH13* in 39,7%, *CASP8* in *25*%, *CD44*-12,5%, *TIMP3*-2%, *ESR1*-9% and *CADM1*-1%. Cohen’s (κ) fair agreement test has shown a linear positive moderate correlation between PSA level and 4 genes (*RARB, APC, GSTP1*, and *CDH13*), and a significant linear positive moderate correlation between Gleason Score and 9 methylated genes (GSTP*1*, *RARB*, *RASSF1*, *APC, CDH13*, *CASP8, IMP3*, *ESR1*, and *CADM1),* except for gene CD44. TMPRSS2-ERG fused transcript was detected in 32 out of 88 (36,4%) of the samples. There were no significant differences between patients with/without detected TMPRSS2-ERG fusion related to Gleason score and PSA level. In conclusion, additional studies need to be performed in appropriately powered clinical cohorts that will define the positive diagnostic meaning of TMPRSS2-ERG fusion transcript, point to the sites in the genome that are under the influence of DNA methylation, in that manner offering additional information for further management of the CaP disease.

## INTRODUCTION

Prostate cancer (CaP) is the most commonly detected cancer and the second leading cause of death in males from Western countries [1]. The major risk factors for CaP are the aging of the male population due to life span prolongation and the spread of the Western-modern lifestyle [1]. Aldo, several diagnostic methods (prostate-specific antigen test (PSA Test), transrectal ultrasonography, and magnetic resonance [2]) have been used as markers for detection of the CaP, still, CaP remains an escalating international health problem. The diagnosis of early-stage CaP is imperative for its successful management since the inevitable emergence of androgen insensitivity in late-stage tumors leads to significant mortality. Nowadays, there is solid evidence that androgens influence the etiology of CaP. supporting this claim is the fact that this type of carcinoma does not occur among eunuchs and men who have been castrated, and the androgen ablation leads to remission of this disease [3]. The care of patients after their initial diagnosis and treatment is difficult due to the lack of specific biomarkers [4].

PSA as the most widely used tumor marker for the detection of prostate carcinoma on its own or combined with its derivates can be used for screening and to detect recurrences before the development of clinical disease, its baseline values do not allow prognostic prediction of disease behavior [4]. Other markers are used for diagnostic purposes at a greater or minor success rate. However, only about half of patients diagnosed will develop significant symptoms, and <20% will die from their disease.

TMPRSS2-ERG fusion transcript leads to the androgen induction of ERG proto-oncogenes expression representing a high presence of oncogenes alteration among prostate tumor cells. This transcript has a significant incidence in human prostate cancer and can be detected in blood and tissue and also non-invasively in urine.

The genes undergoing methylation present promising biomarkers recently investigated on aberrant DNA promoter hypermethylation [5]. During the early phases of tumorigenesis, they could be potentially used as markers for identifying individuals at increased risk of developing malignancy, or for aiding in the diagnosis of early malignancy, those genes undergoing methylation during the progression of malignancy could potentially be used as prognostic markers [5]. This epigenetic change occurs widely in various types of cancerous cells and always affects the same promoter regions [6]. Hypermethylation can also correlate with pathological grade or clinical stage, and contribute to the invasiveness, metastasis, and/or androgen independence of prostate tumors. This research aimed to analyze the presence of the TMPRSS2-ERG fusion transcript and DNA methylation in patients with CaP, as a baseline database for further genetic examination.

## MATERIALS AND METHODS

The research study was implemented at the University Clinic of Urology – Skopje in cooperation with the Institute for Pathological Anatomy – Skopje, the Clinical Biochemistry at the Faculty of Medicine-Skopje; and the Research Centre for Genetic Engineering and Biotechnology “Georgi D. Efremov” at the Macedonian Academy of Sciences and Arts (MASA) in two years. The study cohort consisted of 88 randomly selected prostate cancer patients. The inclusion criteria were as follows: PSA>4ng/ml and positive rectal toucher (suspect digital-rectal examination); men with prostate cancer who were between the ages of 40 and 85, with sociodemographic characteristics being irrelevant. This study excluded patients with coagulopathies, more serious cardiovascular diseases, dementia, severe general and locoregional disease, incurable conditions, and other types of malignancy. The study was approved by the Ethics Committee at the Medical Faculty “Ss Cyril and Methodius” University – Skopje.

### Methods

Extracted RNA or DNA from the prostate biopsy or prostate tissue was used for all molecular analyses. From the histological test of the prostate tissue, we have obtained the results regarding the Gleason score, and we have measured the PSA level from the blood in all 88 samples.

Prostate tissue samples were obtained by biopsy using standard techniques and protocols, which entail at least 10–12 “core” biopsies (plus one or two cylinders) measuring 10–20 mm in length. These biopsies are taken from the prostate’s apex, base, and middle peripheral zone (6–8 from each side of the prostate). A second cylinder was removed for molecular analysis from the suspicious area.

Before beginning the additional analysis at the laboratory at MASA the cylinder was placed and immersed in a 1,5 ml Eppendorf tube filled with liquid nitrogen. It was then snap-frozen and maintained at −80° C. The biopsy process in that area was regularly repeated if a particular sample of the bioptic material was insufficient (insufficiently long). To stop tissue autolysis, the prostate tissue was immediately placed in a container containing 10% formalin after the biopsy procedure. The material was also treated with four distinct reagents, after which it was cut, molded with paraffin, and finally processed with hematoxylin and eosin colors for histopathology and a “Gleason” evaluation. The tissue from the most likely cancerous zone was removed, and the same process was followed for its preparation and analysis as for the biopsy specimen. The sample from the operative material was obtained right after the prostatectomy. In the course of the TMPRSS2-ERG fusion molecular analysis, roughly 10 mg of each tissue sample was used for total RNA extraction. This was done using a commercial kit called the MagCoretriXact RNA Kit and an automated platform called MagCore^®^, RBC Bioscience, Taiwan, which included DNase treatment for DNA elimination. Real-time PCR analysis was used, to detect the mutant and wild-type alleles. TaqMan^®^ fusion assay, TaqMan^®^ gene expression assay, and KiCqStart One-Step Probe RT-qPCR Kit (Sigma-Aldrich, Missouri, USA) were utilized. Using VIC dye to detect the control transcript and FAM dye to detect the fusion, the fluorescent RT-PCR reaction was carried out on 7500 Fast Real-Time PCR Systems. The manufacturer’s recommended cycling conditions and threshold levels were implemented.

The control gene expression assay produced a 79bp fragment containing exons 7–8 of the TMPRSS2 gene, whereas the fusion produced a 106bp fragment containing TMPRSS2 exon 1 and ERG exon 4. The TMPRSS2-ERG exon1–4 isoform can be detected using this duplex assay, with a detection limit of 0.05%.

For DNA methylation analyses, total DNA extraction was performed on the same MagCore^®^ platform (Mag-Core^®^, RBC Bioscience, Taiwan) using a commercial kit MagCore^®^ Genomic DNA Tissue Kit. For analysis of the tumor suppressor genes methylation in the prostate cancer tissues, a SALSA MS-MLPA Probemix assay ME001 (MRC Holland, Amsterdam) kit was used. With this assay, we simultaneously analyzed methylation and copy number changes of 26 genes including *TP73, CASP8, VHL, RARB, MLH1, RASSF1, FHIT, APC, SLC9A2-2, ESR1, CDKN2A, CDKN2B, DAPK1, KLLN, PTEN, CD44, GSTP1, ATM, CADM1, CDKN1B, CHFR, BRCA2, CDH13, HIC1, BRCA1,* and *TIMP3*. Coffalyser.NET software was used for the data analysis. If the methylation dosage ratio, of the digested sample compared with the undigested sample, was ≥0.10 (which corresponds to 10% of DNA methylation), we considered promoter methylated [7].

### Statistics analysis

Version 22.0 of the SPSS software program for Windows was used to statistically analyze the data (SPSS, Chicago, IL, USA). The qualitative series were presented as absolute and relative numbers after the computation of coefficients of relations, proportions, and rates. Both dispersion and central tendency measures (mean, median) were used to analyze quantitative series (standard deviation, standard error). The Shapiro-Wilk W test was used to determine if the distribution was normal. Spearman Rank Order Correlations were used to determine the direction and strength of the association between two variables. To assess agreement, TMPRSS2-ERG RT-PCR and genome-wide DNA methylation were compared using Cohen’s kappa (κ). The statistical significance was ascertained using a two-sided analysis with a significance level of p<0,05.

## RESULTS

In the study sample of 88 (100%) patients with prostate carcinoma, the average age was 69,1±7,1 year with min/max age of 50/85 years and 50% patients younger than 70 years for Median IQR=70 (64–74) (Table 1). The average value of PSA was 29,9±34,40ng/ml, with a min/max value of 4,3/152 ng/ml and Median IQR=14,3 (9,1–38). We determined a Gleason Score (Gleason Major & Gleason Minor) for every patient in the study cohort (Table 1). The average values of Gleason Major were 3,4±0,6, whereas those of Gleason Minor were 3,5±0,6 with min/max of 3/5 and Median IQR=3 (3–4) for both scores. The average Gleason Score was 6,9±0,9 with a min/max value of 6/9 and 50% where this score was lower than 7 for Median IQR=7 (6–7). TMPRSS2-ERG fused transcript was detected in 36,4% (32 out of 88) of the patients with CaP (Table 1). For patients with detected TMPRSS2-ERG fused transcript, the PSA level was 28,1±26,6ng/ml with min/max 4,3/116. Among 50% of these patients, the PSA level was lower than 19ng/ml for Median IQR=19 (9,6–40,1). For patients where the TMPRSS2-ERG transcript was not detected, the average PSA level was 30,9±38,1ng/ml with min/max 4,5/152 and 50% of them with a level lower than 14 ng/ml for Median IQR=14 (8,5–32). We didn’t find a significant difference between patients with prostate cancer who were with/without detected TMPRSS2-ERG fused transcript related to PSA level (Mann-Whitney U test: Z=−0,725; p=0,4685). Average Gleason Major, Gleason Minor, and Gleason Score for patients with/without detected TMPRSS2-ERG fused transcript was 3,41±0,56 vs. 3,43±0,56; 3,50±0,57 vs. 3,48±0,65; 6,91±0,82 vs. 6,91±0,92 respectively. We also found no significant differences between patients with/without detected TMPRSS2-ERG fused transcript related to Gleason Major (Mann-Whitney U test: Z=0,1728; p=0,8628), Gleason Minor (Mann-Whitney U test: Z=−0,3456; p=0,7296) as well as Gleason Score (Mann-Whitney U test: Z=−0,1475; p=0,8827).

**Table 1.** Qualitative TMPRSS2 – ERG RT-PCR among patients with prostate carcinoma

**Sample**	**Age**	**PSA**	**GMa^1^**	**GMin^2^**	**GS^3^**	**TMPRSS2–ERG Real-Time PCR**	**Sample**	**Age**	**PSA**	**GMa^1^**	**GMin^2^**	**GS^3^**	**TMPRSS2–ERG Real-Time PCR**
**Ca P - 1**	62	9,7	3	3	6	Negative, type	**Ca P – 45**	73	14	3	3	6	Negative, type
**Ca P – 2**	63	32	4	4	8	Negative, type	**Ca P – 46**	63	100	4	4	8	Negative, type
**Ca P – 3**	74	16,5	4	5	9	Negative, type	**Ca P – 47**	77	7,4	3	3	6	Negative, type
**Ca P – 4**	74	84	3	4	7	Negative, type	**Ca P – 48**	76	65	4	3	7	Positive, type *
**Ca P – 5**	64	8,28	3	3	6	Negative, type	**Ca P – 49**	60	44	3	4	7	Positive, type *
**Ca P – 6**	71	7,38	3	4	7	Positive, type *	**Ca P – 50**	80	45,3	3	4	7	Positive, type *
**Ca P – 7**	78	17	3	4	7	Negative, type	**Ca P – 51**	64	12	3	3	6	Negative, type
**Ca P – 8**	64	45	4	4	8	Positive, type *	**Ca P – 52**	74	10,2	4	3	7	Negative, type
**Ca P - 9**	72	20,1	3	3	6	Negative, type	**Ca P – 53**	85	58	3	3	6	Negative, type
**Ca P – 10**	64	4,33	3	3	6	Positive, type *	**Ca P – 54**	75	25	3	4	7	Positive, type *
**Ca P - 11**	59	9,15	4	3	7	Positive, type *	**Ca P – 55**	67	20,5	4	3	7	Negative, type
**Ca P - 12**	70	8,53	3	3	6	Negative, type	**Ca P – 56**	72	9,28	3	3	6	Positive, type *
**Ca P - 13**	74	14	4	3	7	Negative, type	**Ca P – 57**	64	37	3	4	7	Positive, type *
**Ca P - 14**	61	4,5	3	3	6	Negative, type	**Ca P – 58**	68	13,3	3	3	6	Negative, type
**Ca P - 15**	55	63,5	4	4	8	Negative, type	**Ca P – 59**	61	6,1	3	5	8	Negative, type
**Ca P - 16**	85	24	3	3	6	Positive, type *	**Ca P – 60**	69	8,2	3	3	6	Negative, type
**Ca P - 17**	70	9,4	3	3	6	Positive, type *	**Ca P – 61**	71	14,5	4	4	8	Negative, type
**Ca P - 18**	78	15,5	5	3	8	Positive, type *	**Ca P – 62**	77	68,9	4	3	7	Negative, type
**Ca P - 19**	69	18	3	3	6	Positive, type *	**Ca P – 63**	77	25	4	3	7	Positive, type *
**Ca P - 20**	71	9,7	3	4	7	Negative, type	**Ca P – 64**	70	12	3	4	7	Negative, type
**Ca P - 21**	67	18	3	3	6	Negative, type	**Ca P – 65**	65	42,1	4	4	8	Positive, type *
**Ca P – 22**	74	70	4	4	8	Negative, type	**Ca P – 66**	62	26,2	3	3	6	Positive, type *
**Ca P – 23**	69	22,3	4	4	8	Negative, type	**Ca P – 67**	70	10,6	3	3	6	Negative, type
**Ca P - 24**	68	9,44	4	3	7	Negative, type	**Ca P – 68**	78	102	3	4	7	Negative, type
**Ca P - 25**	70	45,8	4	5	9	Negative, type	**Ca P – 69**	74	6,1	3	4	7	Positive, type *
**Ca P – 26**	67	12,7	3	3	6	Negative, type	**Ca P – 70**	58	10,3	3	3	6	Positive, type *
**Ca P - 27**	62	24	4	3	7	Negative, type	**Ca P –71**	65	5,8	3	4	7	Positive, type *
**Ca P - 28**	64	94	4	4	8	Positive, type *	**Ca P – 72**	70	11,7	3	4	7	Negative, type
**Ca P – 29**	50	12,2	3	3	6	Positive, type *	**Ca P – 73**	70	14	3	3	6	Positive, type *
**Ca P – 30**	66	6,1	3	3	6	Negative, type	**Ca P –74**	70	9,72	4	4	8	Positive, type *
**Ca P - 31**	53	70	3	4	7	Positive, type *	**Ca P – 75**	72	14,3	4	3	7	Negative, type
**Ca P - 32**	69	116	4	5	9	Positive, type *	**Ca P - 76**	77	20	4	3	7	Positive, type *
**Ca P - 33**	71	7	4	3	7	Negative, type	**Ca P - 77**	81	5,42	3	3	6	Negative, type
**Ca P - 34**	63	6,2	3	3	6	Negative, type	**Ca P – 78**	78	38	4	3	7	Positive, type *
**Ca P – 35**	64	148	4	3	7	Negative, type	**Ca P – 79**	75	10,4	3	4	7	Positive, type *
**Ca P – 36**	77	150	4	3	7	Negative, type	**Ca P - 80**	68	11	3	3	6	Negative, type
**Ca P – 37**	65	4,7	3	4	7	Negative, type	**Ca P - 81**	69	23	4	4	8	Positive, type *
**Ca P – 38**	61	7,1	3	4	7	Negative, type	**Ca P - 82**	70	7,8	3	3	6	Positive, type *
**Ca P – 39**	80	90	5	4	9	Negative, type	**Ca P - 83**	55	11,6	3	3	6	Negative, type
**Ca P – 40**	62	7,62	5	3	8	Negative, type	**Ca P – 84**	75	11	3	3	6	Positive, type *
**Ca P – 41**	72	8	3	4	7	Negative, type	**Ca P – 85**	62	7,5	3	3	6	Negative, type
**Ca P – 42**	76	21,3	3	3	6	Negative, type	**Ca P – 86**	70	90	3	4	7	Negative, type
**Ca P – 43**	77	8,5	4	5	9	Negative, type	**Ca P – 87**	54	8,5	3	3	6	Negative, type
**Ca P – 44**	59	152	4	4	8	Negative, type	**Ca P – 88**	72	43	3	5	8	Negative, type

1 GMa - Gleason Major

2 GMin - Gleason Major

3 GSMa - Gleason Score

We found that TMPRSS2-ERG fused transcript of patients with prostate cancer, non-significantly positively correlated with age (R_(88)_=0,016; p=0,880), PSA value (R_(88)_=0,077; p=0,472) as well as Gleason Score (R_(88)_=0,008; p=0,934). We analyzed genome-wide DNA methylation profiles of 26 tumor suppressor genes in CaP tissue from all 88 patients. Expression was not detected in 61,54%, (16 out of 26) analyzed genes (TP73, VHL, MLH1, FHIT, CDKN2A, CDKN2B, DAPK1, KLLN, PTEN, ATM, CDKN1B, CHFR, BRCA2, HIC1, BRCA1 and SLC9A2). Cohen’s κ determines fair agreement between TMPRSS2 – ERG RT-PCR and: a) GSTP1-1 (p=0,029) and b) CD44-1 (p=0,044). None to slight agreement was found between TMPRSS2 – ERG RT-PCR and all other analyzed genes (Table 2).

**Table 2.** Qualitative genome-wide DNA methylation among patients with prostate cancer and gene correlation TMPRSS2 – ERG RT-PCR, age, PSA and Gleason Score

**DNA methylation Genes**	**N (%)**	**Cohen’s kappa (κ)^1^**	**Correlation**		
			**Age**	**PSA**	**Gleason Score**
**CASP8-3**	22 (25,00%)	κ = 0,158 [95% CI (0,049 – 0,360)]; *p* = 0,125	r (88)=0,124; p=0,251	r (88)=0,199; p=0,062	r (88)=0,369; p=0,0003*
**RARB-1**	39 (44,32%)	κ = 0,179 [95% CI (0,017 – 0,377)]; *p* = 0,089	r (88)=0,085; p=0,432	r (88)=0,335; p=0,001*	r (88)=0,399; p=0,0001*
**RASSF1-1**	56 (63,64%)	κ = 0,154 [95% CI (0,021 – 0,331)]; *p* = 0,094	r (88)=0,251; p=0,018*	r (88)=0,171; p=0,111	r (88)=0,274; p=0,009*
**APC-2**	37 (42,04%)	κ = 0,121 [95% CI (0,092 – 0,318)]; *p* = 0,253	r (88)=0,148; p=0,167	r (88)=0,364; p=0,004*	r (88)=0,364; p=0,0004*
**ESR1-1**	8 (9,09%)	κ = 0,112 [95% CI (−0,232 – 0,022)]; *p* = 0,141	-	-	-
**CD44-1**	11 (12,50%)	κ = 0,171 [95% CI (0,000 – 0,357)]*; p* = 0,044*	r (88)=0,205; p=0,056	r (88)=0,064; p=0,552	r (88)=0,122; p=0,259
**GSTP1-1**	47 (53,41%)	κ = 0,219 [95% CI (0,038 – 0,414)]*; p* = 0,029*	r (88)=0,181; p=0,094	r (88)=0,274; p=0,009*	r (88)=0,339; p=0,001*
**CADM1-1**	1 (1,14%)	κ = 0,023 [95% CI (−0,067 – 0,000)]*; p* = 0,447	-	-	-
**CDH13-1**	35 (39,77%)	κ = 0,158 [95% CI (−0,045 – 0,363)]; *p* = 0,138	r (88)=0,154; p=0,152	r (88)=0,284; p=0,007*	r (88)=0,266; p=0,012*
**TIMP3-1**	2 (2,27%)	κ = 0,045 [95% CI (−0,110 – 0,000)]; *p* = 0,280	-	-	-

1 Agreement between TMPRSS2 – ERG RT-PCR and selected gene

* significant for p<0,05

We have found a significant linear positive weak correlation only between the RASSF1-1 gene and the age of CaP patients (R_(88)_=0,251; p=0,018), (Table 2). Regarding the PSA level, our analysis shows a significant linear positive moderate correlation between the PSA level and 4 genes: RARB-1 (R_(88)_=0,335; p=0,001); APC-2 (R_(88)_=0,364; p=0,004); GSTP1-1 (R_(88)_=0,274; p=0,009) and CDH13-1 (R_(88)_=0,284; p=0,007) (Table 2).

When we correlated the Gleason score and 10 methylated genes, we found a significant linear positive moderate correlation between the Gleason Score and 9 methylated genes (Table 2), except gene *CD44* for which the correlation was positive but not significant for p>0,05, due to the small number of expressions.

## DISCUSSION

Prostate cancer is the most common male malignancy and the second leading cause of cancer-associated death for men in the developed world [8]. The introduction of the prostate-specific antigen (PSA) test in the late 1980s has resulted in a marked increase in the diagnosis of prostate cancer but has not subsequently led to a dramatic reduction in death from prostate cancer [9]. A large effort in searching for markers that may more accurately identify aggressive prostate cancer, however, has yet to yield a widely adopted clinical assay. Despite the current lack of clinically applied prostate cancer biomarkers capable of increasing prognostic accuracy, there has been a heavy focus on the development of molecular markers of a genetic, expression, and proteomic nature, such as DNA methylation and TMPRSS2-ERG fusion [10].

TMPRSS2-ERG gene family fusions were the first commonly occurring gene fusions discovered in epithelial malignancies [11]. The most common of these fusions, TMPRSS2-ERG, can occur in approximately 50% of prostate cancer cases [12]. This fusion event most often arises from the deletion of an interstitial fragment of chromosome 21, resulting in the androgen-responsive promoter and 5′ end of *TMPRSS2* driving the expression of the ERG oncogene [12]. Fusion frequency more than 50% has been cited also by Soller and his associates, but this high discrepancy is due to the small number of cases (n=18) and/or the use of included PCR [13]. Many types of research that have been done, concluded that TMPRSS2-ERG fusion disorder can be detected through the RT-PCR methods in prostate cancer patients- tissue, blood, and urine [14]. In our study, the TMPRSS2-ERG fusion transcript was detected in 32 (36,4%) out of 88 patients with CaP. Indicating that our results are in concordance with the findings in several European research centers, in which a significant proportion of TMPRSS2-ERG genetic rearrangements and alterations have been confirmed. Mehra et al.found that TMPRSS2-ERG mRNA expression was present in 10 of 27 (37%) of hormone-refractory metastatic prostate cancer patient samples [15], and in the research of cases defined as localized prostate cancer, Liu B et al. concluded that TMPRSS2-ERG fusion was positive in 28.0% (14/50)[16]. The presence of the TMPRSS2-ERG fusion was detected in 34 out of 108 samples in Czech patients, (31.5%) analyzed by the RT-qPCR method with UPL probes quantifying mRNA transcripts, with the possibility to measure the expression level as well [17]. The TMPRSS2:ERG rearrangement can be found in about one-third of prostate cancers. Saramaki et al. detected that 50 out of 150 (33%) of the prostatectomy specimens overexpressed ERG and had TMPRSS2:ERG rearrangement, and in that subgroup, they identified prostate cancer patients with good prognosis.[18].

In our research, there were no significant differences between patients with/without detected TMPRSS2-ERG fusion transcript related to Gleason score as well as no difference between patients with prostate cancer who were with/without detected TMPRSS2-ERG fused transcript related to PSA level. In Slovenian patients, the association between *TMPRSS2:ERG* fusion and Gleason score before operation was not significant. [19]. In a study of 99 Turkish patients who underwent radical prostatectomy because of prostate cancer, PSA and Gleason were analyzed with independent samples t-test to predict TMPRSS2-ERG fusion, and none of these variables were found to be statistically significant. [20]. Many studies are conducted to determine if the TMPRSS2-ERG fusion transcript can be associated with the pathogenesis of prostate carcinoma and if it can be used in the early detection, prediction as well as prognosis of this disease [21, 22, 23, 24, 25, 26]. Conflicting results about the prognostic value of TMPRSS2 fusions have also arisen, with some reports claiming a positive association with clinicopathologic features such as disease stage and metastases as well as disease recurrence [27], others have found no association with these variables [28, 29].

Epigenic modifications such as DNA methylation, regulate gene expression via chromatin remodeling. Multiple genome-wide DNA methylation is important in maintaining the integrity of the genome, and studies in prostate cancers have identified genes that are differentially methylated in malignant tissue compared with non-malignant tissue, or in association with hormone receptor status, or tumor recurrence. Aberrant DNA methylation is a common anomaly in prostate cancer that results in increased methylation at the promoters of tumor suppressor genes [30, 31, 32], that involves covalent bonding of a methyl group specifically to the dinucleotide CpG, which is catalyzed by the family of enzymes known as DNA methyltransferases (DNMTs). Numerous studies have supported the important role of DNA methylation in carcinogenesis, demonstrated that can act as a disease biomarker, and have shown that targeting aberrant DNA methylation is a promising cancer therapeutic[19,33,34].

Some of the commonly reported hypermethylated genes in prostate cancer include *GSTP1* *RASSF1A* and *APC. GSTP1* is associated with the early development of prostate cancer, whereas *RASSF1A* and *APC* have been reported to cluster more frequently in higher-grade and later-stage malignancies[19, 33,34]. In our research, we have analyzed the genome-wide DNA methylation profile of 26 tumor suppressor genes in the prostate cancer tissue of 88 patients. We have found that 10 out of the 26 genes (38.5%) were methylated. The most frequently methylated gene was RASSF*1,* found in - 63,6%, *GSTP1* in 53,4 %, *RARB* in 44,32 %, and *APC* in 42 %, of the analyzed patients. Additionally, we have found a high frequency of methylation in *CDH13, CASP8*, and *CD44* (detected in 39,7%, 25%, and 12,5%, respectively.) A smaller percentage was found for 3 genes (TIMP3-2%, ESR1-9%, and CADM1-1%). Our results are in concordance with the results of Harden et al. which detect *GSTP1* methylation in 11 out of 15 cases of limited prostate cancer and 0 of 14 of benign disease (2-sided Fisher’s exact test, p <0.0001) [35]. Also, we have similar results to the research of Singal et al. which examined the methylation status of *GSTP1, RARB, CD44, ECAD, RASSF1A, and EDNRB* genes in 81 prostate cancer. Methylation frequencies in prostate cancer were 72% for *GSTP1*, 40% for *RARB*, 72% for *CD44*, 61% for *ECAD,* 49% for *RASSF1A*, a72% and 62% for EDNRB [36,37].

Regarding the association between mutilated genes and Gleason Score / PSA our analysis shows a significant linear positive moderate correlation between PSA level and *RARB* (R_(88)_=0,335; p=0,001); *APC* (R_(88)_=0,364; p=0,004); *GSTP1 (*R_(88)_=0,274; p=0,009) and *CDH13* (R_(88)_=0,284; p=0,007), and between Gleason Score and all analyzed genes with exception of gene *CD44* for which the correlation was positive but not significant for p>0,05, that may be due to the small number of expressions. Lissette Delgado-Cruzata et al detected that there were no significant associations between the studied DNA methylation markers tumor stage, and preoperative PSA levels but DNA methylation levels in tumor tissue were statistically significantly higher for cases with Gleason score ≥7 compared with those with Gleason score <7 for *GSTP1*, *p16* (*CDKN4A*), and *APC* [38]. Zhou M et al. investigated whether *GSTP1* methylation correlated with Gleason grade and tumor volume in prostate biopsies. *GSTP1* methylation was positive in 83 of 96 cancer parts (86.5%). Quantitative *GSTP1* methylation correlated with Gleason grade, demonstrating higher *GSTP1* methylation in higher Gleason grade tumors (Spearman correlation coefficient 0.377, p < 0.0001) [39]. Additional studies that integrate TMPRSS2-ERG gene fusion and genome-wide DNA methylation are essential in gaining novel reflections and conclusions about prostate cancer. They demonstrate the potential of identifying epigenetically regulated genes and gene fusion transcripts that are involved in prostate cancer initiation or progression and therefore could be biomarkers of prognosis and diagnosis of the disease.

Studies of this nature must be performed in appropriately powered clinical cohorts with the relevant patient samples and should define the positive diagnostic meaning of TMPRSS-ERG fusion transcript and produce an unbiased, high-quality ‘roadmap’ of all sites in the genome that are under the influence of DNA methylation. These studies will offer additional information, in the decision-making process on the further management of prostate cancer.
